# Veracity of Homæopathists

**Published:** 1848-08

**Authors:** 


*Veracity of Homœopathists.*—It is a common remark, that "figures cannot lie." This remark, however, was never intended to cover the veracity of those by whom the figures are made. Lying by means of figures is as feasible as it is common with those, who, to promote their own base purposes, are ready to avail themselves of any method of deception which promises success; and no sophistry is so well calculated to deceive as false statistics. Most of our readers, probably, are aware, that in an article by Dr. Forbes, in one of the expiring numbers of the late British and Foreign Medical Review, (an article which impaired not a little the respect of the profession for the good sense of the author,) the reports of Dr. Fleishmann, of the results of homœopathic treatment, at the hospital in Vienna, were cited as exhibiting a fair test of this method of practice. It turns out, as we learn by the London Lancet, that these statistical statements, were arrant forgeries. An examination of the books of the establishment has led to their detection. How far the records themselves are false, of course cannot so well be ascertained. They were made at a homœopathic establishment, under the direction of one who is dishonest enough to give a false representation of the results of their analysis; hence, it is fair to presume, that if the matter could be sifted to the bottom, it would be found that the case is still worse for homœopathy than it even now is proved to be.

A somewhat similar trick has lately been attempted in the city of New-York. The trustees of the "Homœopathic Dispensary Association" published a card, addressed to the public, setting forth, in figures, the results of homœopathy at an orphan institution, as contrasted with results, at the same institution, while the sick received regular medical attendance. Dr. James McCune Smith, of New-York, was induced to investigate the matter, and he proves from the same source whence the figures appearing on the *card* were taken, that, so far from figures establishing the superior success of Homœopathy, the odds are more than double against it. We refer those who have any curiosity to examine the matter, to our respected contemporary, the Annalist, (No. 18, vol. 11,) which contains the card, and the article by Dr. Smith, together with some excellent remarks by the editor. That the Trustees who have lent their names on the *card* are guilty of a deliberate intention to deceive the public, may not be true, but it was certainly their duty to ascertain that there could be no room for doubt as to the correctness of the statistics, before consenting to endorse them.

It is not surprising that they who have enrolled themselves as Homœopathists with a view to making the most of it as a pecuniary speculation upon public credulity, should resort to these, and other equally dishonest means of furthering their ends. Not less would be expected from the character of their motives; it is carrying out the spirit which, as Dr. Lee has abundantly showed, actuated the founder of the imposition. Many of them have succeeded in their objects, and are now ready to laugh at the folly which has filled their pockets.

The latter consolation will not remain for those who have been duped. We really cannot but commiserate the few unfortunate gentlemen, and more particularly, the not so few philanthropic ladies, who have felt it to be their duty to go from house to house, endeavoring to persuade invalids to dismiss their medical advisers, and try a homœopathist. This zeal, which thus prompts them to overstep the bounds of delicacy, and we might add, decency, is pretty good evidence that they do not feel quite secure in their confidence in the merits of their cause. It savours of the plan pursued by the fox, in the fable, who had lost his caudal appendage. Mortification, more than any other species of misery, likes company. But this subject is too grave for ridicule. If it concerned merely mortified pride, or the loss of a few dollars, it would be quite a different matter from what it really is. It is a subject involving health and life. If persons in the independent exercise of their judgment, elect a course calculated to jeopardize these most valued of all temporal blessings, they may be compassionated, but perhaps are not culpable. It is otherwise with those who officiously obtrude upon the sick or their friends, and taking advantage of the anxiety and weakness which are incident to disease, succeed in diverting, or, at least, impairing the resources ot medical art. We envy not the moral perceptions which do not recognize the fearful responsibility which such conduct involves; nor the conscience which is insensible to the reproaches (albeit unuttered) which are breathed in the unavailing regrets of those who mourn for departed friends.

